# The role of diet and food supplements in infertility management in the Eastern Mediterranean Region: A narrative review

**DOI:** 10.18502/ijrm.v23i1.18188

**Published:** 2025-03-21

**Authors:** Kassandra Said Fares, Georges Hani Abi Tayeh, Emile Roger Whaibeh, Romy Jihad Louis, Lina Yasser Jaalouk, Yara Matar Matar, Myriam Andraos Mrad

**Affiliations:** ^1^Higher Institute for Public Health, Saint Joseph University of Beirut, Beirut, Lebanon.; ^2^Saint Joseph Fertility Center, Beirut, Lebanon.; ^3^Health and Environment Response Agency, Hera, Lebanon.; ^4^Unit of Obstetrics and Gynecology and Fertility, Hotel-Dieu de France Hospital, Beirut, Lebanon.; ^5^Faculty of Medicine, Saint Joseph University of Beirut, Beirut, Lebanon.; ^6^Public Health Department, Faculty of Health Sciences, University of Balamand, Beirut, Lebanon.

**Keywords:** Infertility, Diet, Vitamins, Antioxidants, Assisted reproductive techniques.

## Abstract

According to the World Health Organization, 17.5% of the population suffers from infertility. This demonstrates the critical need to expand access to high-quality reproductive care and increase our understanding of the factors that contribute to this issue. This review aims to summarize findings and gaps in the literature regarding diet-related factors and infertility among the Eastern Mediterranean couples poorly studied compared to other populations. The literature search was conducted using PubMed and Scopus databases from January 2012-July 2023. In total, 112 papers described the influence of diet and supplementation on natural and artificial reproductive outcomes in the Eastern Mediterranean Region and were found eligible for the review synthesis. For men, a diet rich in red meat, fatty foods, whole grains, and low in fish, poultry, low-fat dairy products, and vegetables have a positive effect on sperm count. For women, maintaining a healthy weight is crucial for their fertility. Overall, various vitamins and supplements significantly enhance gametes quality, hormonal balance, and antioxidant capacity, despite the results not being consistent across all studies. The findings highlight distinct dietary patterns that mitigate or exacerbate infertility risks, considering macro and micronutrients. Studies unevenly describe potential risk factors, underscoring the need for further exploration across diverse populations.

## 1. Introduction

Infertility is a growing public health concern that can have a significant impact on the well-being of young couples. According to the World Health Organization in 2023, 1 in 6 adults globally, or 17.5% of the population, suffer from infertility (1). This demonstrates the critical need to expand access to high quality, reasonably priced in vitro fertilization (IVF) care for those who require it, as well as increase our understanding of the factors contributing to this issue.

Although the association between a person's diet and their ability to reproduce is becoming more widely acknowledged, some researchers contend that there is currently no official guidance available for couples of reproductive age (2). For instance, the “Westernized” (carbohydrates, fats, and sodium), “Mixed” (fruits, vegetables, and sugars), or “Neo-Mediterranean” (proteins, fibers, and calcium) dietary patterns identified among the Lebanese population did not correlate with the neonatal outcomes of pregnant women (3). A cross-sectional study on the same population attributed low levels of adherence to the Mediterranean diet to > 50% of fertile participants (4). A diet that includes eating a lot of whole grains, vegetables, fruits, fish, and mono- or polyunsaturated oils has been linked to increased semen quality in men and improved fertility in women (5). The Mediterranean diet is favorable because it is distinguished by a low intake of red meat and simple sugars and a high intake of fruits, vegetables, olive oil, unrefined carbs, low-fat dairy and poultry, and oily fish (6).

Micronutrient supplementations show potential in improving fertility. A retrospective study on 1000 pregnant women suggested an association between vitamin B9 intake and neonatal results (7). Men who consumed more vitamin C, vitamin E, folate, and zinc showed less fragmentation of sperm DNA, according to a study conducted in the United States (8). An inadequacy in vitamin D levels may be harmful to fertility. However, taking vitamin D supplements while having already reached sufficiency may not offer any extra benefits (2).

The Eastern Mediterranean Region (EMR) encompasses 22 countries located in Asia and Africa with a population of more than 735 million by 2020. The region suffers from a dearth of information regarding infertility (9). This is primarily due to inadequate funding for research and insufficient resources available to various governmental and nongovernmental organizations. Moreover, there is also a chance that publications in the Middle East and North Africa are primarily published in regional languages, making them difficult to get identified in online resources. A systematic review with a meta-analysis of the infertility prevalence survey found that while demographic infertility in this region is significant (22.6%), clinical primary infertility is low (3.8%) (10).

**Figure 1:**
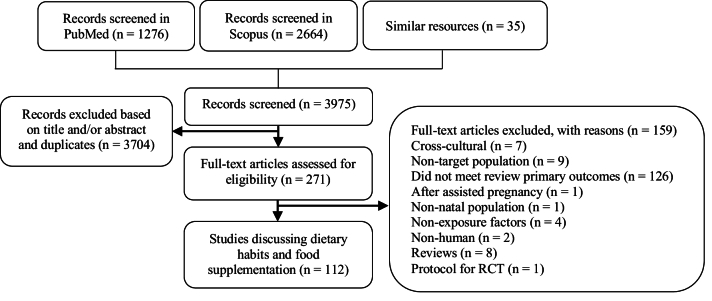
Flow chart for the included studies in the review.

The current review aims to discuss the impact of dietary habits, including food types and dietary supplements, on fertility in the EMR. PubMed and Scopus databases were used for the literature search. The search was restricted to publications between January 2012 and July 2023. Moreover, this review was conducted with limited search words and is subject to limitations, including potential omissions of references, and should not be interpreted as an intentional omission, as it does not adhere to the systematic review methodology. Only 112 publications met the inclusion criteria and were considered suitable for this review (Figure 1), as they directly covered dietary habits and food supplementation's effects on reproduction in the EMR. The studies were mainly conducted in the following countries: Iran (66%), Iraq (20.5%), and Pakistan (5.3%) (Table I). Only 7 countries of the EMR published data matched the inclusion criteria of the review.

**Table 1:** Number of articles published by EMR countries studying the effect of diet-related risk factors among infertile participants

**References**	**Risk factors**	**Countries**	**Number of papers**
**(11–28)**	Dietary habits	Iran	17
		Iraq	1
**(29–32)**	Lipids	Iran	4
**(33–87)**	Vitamins	Iran	34
		Iraq	11
		Pakistan	4
		Egypt	4
		Jordan	1
		Saudi Arabia	1
**(88–113)**	Antioxidants	Iran	15
		Iraq	7
		Egypt	1
		Jordan	1
		Tunisia	1
		Pakistan	1
**(114–122)**	Others (L-carnitine, spirulina)	Iraq	4
		Iran	4
		Pakistan	1
EMR: Eastern Mediterranean Region			

## 2. Dietary patterns

3 articles covered the relationship between male infertility and dietary habits (11–13). Diets rich in fish, eggs, low-fat dairy, green leafy vegetables, and vegetable oils indicated lower odds of infertility (11), and decreased the risk of reduced sperm motility by 54% (13). On the other hand, diets rich in red meat, processed meat, organ meat, animal fat, refined grains, and high-fat content foods increased the chances of sperm parameter alteration (11, 13). A traditional Iranian diet, containing organ meat, dairy products, saturated fat, fruits and juices, legumes, and sugary items were associated with reduced abnormal sperm concentration by 83% but also suggested lower volumes of ejaculate (12). Another subgroup of asthenospermic men was assessed to justify the positive effect of an antioxidant-rich diet. The consumption of food containing vitamins E, C, and D, zinc, folate, fiber, selenium, and polyunsaturated fats reduced the chances of asthenozoospermia (14).

A positive correlation was proposed between folate and sperm motility, between vitamin E levels and sperm normal shape, and between zinc and both concentration and motility (15). Furthermore, normospermic men were discovered to eat less processed meat and sweets, and more of fruits, vegetables, skimmed milk, chicken, and seafood (16). One study on the index of spermatic DNA fragmentation encouraged consuming ginger (17).

A cohort study (n = 140), conducted in Iran assessed the relationship between women's infertility and dietary habits (18). 3 dietary patterns were identified: the “healthy diet”, the “unhealthy diet”, and “the western diet”. The “healthy diet” included fruits, vegetables, red and white meat, dairy products, legumes, and nuts. The “unhealthy diet” consisted of mayonnaise, butter, oils (solid), and junk food, and the third pattern, named “western diet”, reflected high consumption of sweets and caffeine, potatoes, grains, oils (liquid), fast food, and salt.

No significant association was found between adapting any of these 3 diets and the fertilization rate nor the number of good embryos. However, it was shown that adopting a healthy diet increased the number of eggs retrieved and the percentage of mature oocytes, and it reduced the risk of polycystic ovary syndrome (PCOS) (18, 19). Infertile women were 2.2 times as likely than healthy women to be overweight or obese, hence consuming a diet with higher calorie and limiting physical activity increases the risk of obesity thus infertility (20). In men, one cross-sectional study found a correlation between eating a healthy diet and sperm concentration (21). Targeting more specific food categories in Iraq, a study found a correlation between infertility risk and drinking more tea and carbonated beverages and eating more chicken and fish. Whereas other studies suggested that red meat, corn oil, and olive oil were beneficial for reproductive power (22, 23). As for assisted reproductive technology (ART), the embryological results were inversely associated with sausage, ham, fat, and oil ingestion (23). In particular, IVF outcomes and clinical pregnancy success were better among women following the Iranian traditional medicine-based diet (24). When it comes to the impacts of diets that cause inflammation on fertility, results are conflicting.

Other studies found that diets that increased inflammation were measured through a dietary inflammatory index and were suggested to increase the chances of spontaneous abortion in women (25). Moreover, no correlation between the dietary inflammatory index and the outcomes of ART was found (26). Furthermore, the reduction in anti-Mullerian hormone (AMH) levels is favored by meat and fast-food consumption, while the slowing down of the decline is favored by dairy products, fats, proteins, and carbohydrates (27, 28).

### Lipids

Fatty acids were addressed for their contribution to the reproductive system. Asthenospermia odds were decreased with higher dietary intakes of omega-3 polyunsaturated fatty acids and Docosahexaenoic acid (29). In addition, the administration of omega 3 and omega 6 to oligoasthenospermic (OAT) infertile men showed that the ratio of omega 6 over omega 3 was higher in comparison to the controls. Furthermore, infertile PCOS women who were supplemented with omega 3 underwent a decrease in the luteinizing hormone (LH) and a significant change in the ratio of LH to follicle-stimulating hormone (FSH) (30). On the other hand, the ratio of eicosapentaenoic acid to arachidonic acid was positively correlated with the endometriosis severity in infertile women with endometriosis (31). In contrast to oleic acid, stearic acid and alpha-linolenic acid were negatively correlated with the number of mature eggs and cleaved embryos. Other fatty acids such as arachidonic acid, palmitic acid, and pentadecanoic acid were also associated with the cleavage rate (32).

### Vitamins

#### Vitamin B 

2 studies demonstrated the positive effect of vitamin B9 and vitamin B12 on sperm parameters of infertile men (33, 34). Sufficient levels of folic acid, vitamin B12, and methylmalonic acid improved the count, motility, and morphology of semen (33). Researchers measured the levels of B12 and B9 vitamins along with a dietary assessment of the intake of these vitamins using a food frequency questionnaire and were also able to show that sufficient consumption of both vitamins positively influences sperm parameters (34).

Moreover, measuring vitamins in 18 female participants diagnosed with PCOS before and after metformin therapy found reduced serum levels of vitamin B12 (35). Furthermore, 6 randomized controlled trials (RCTs) assessed the effect of myo-inositol and folic acid supplementation on infertile women; 4 RCTs evaluated the ART results of women with PCOS and 2 RCTs evaluated the outcomes of participants with poor ovarian response (36–41). All trials showed higher numbers of mature eggs and cleaved embryos of good quality in comparison to the control groups. The fertilization rate was statistically significant as compared to the number of retrieved oocytes, the implantation and clinical pregnancy rates which were higher in the intervention groups but did not show a constant significant difference.

#### Vitamin E and C 

2 controlled trials compared the effects of vitamin E and Ceratonia siliqua (Carob) syrup on sperm characteristics, favoring Carob over vitamin E (42, 43). 2 groups of 30 men with either oligozoospermia, asthenospermia, or teratospermia, were given vitamin E capsules or Carob syrup for 90 days (42). In the same year, another 50 idiopathic infertile men administering Carob in the form of 500 mg capsules were tested (43). Moreover, 3 randomized interventional studies conducted in Iran and Iraq discussed the impact of vitamin E combined with other vitamins or trace elements on male fertility (44–46). The first randomly divided 60 individuals into 2 equal groups receiving either selenium and vitamin E or a placebo (44), in the second they added a 5 mg tablet of folic acid into the regimen of the intervention group (45). On the other hand, 32 idiopathic oligospermic men were administered a combination therapy of vitamin E and C, zinc, selenium, and coenzyme Q10 (CoQ10), which significantly increased the sperm motility and the percent of functional and viable sperm concentration (46). Other studies found that vitamin E combined with Docosahexaenoic acid or with clomiphene citrate to oligo- or astheno-zoospermic men improved the sperm's progressive motility and concentration (47, 48).

3 studies assessed the effects of vitamin E on IVF outcomes in women (49–51), yielding inconsistent results. Adding 1500 IU of vitamin E daily clomiphene citrate and metformin treatment in women suffering from clomiphene citrate-resistant PCOS, increased ovulation and endometrial thickness but did not significantly improve pregnancy rate, or follicular metrics (49). Other studies correlated vitamin E levels in the aspirated follicular fluid with the number and morphology of mature eggs and the quality of cleaved and blastulated embryos (50, 51). Similar findings with vitamin C in the follicular fluid of 50 people were reported (52). 2 RCTs on male partners tested vitamin C efficacy. The first found that ascorbic acid therapy improved sperm motility and morphology post-varicocele surgery but not the count (53). Other researchers demonstrated better sperm morphology and higher concentration levels in the inflammatory profiles and semen analysis of supplemented men of wives with recurrent miscarriages (54).

#### Vitamin D 

14 studies addressed the link between vitamin D and female fertility while 19 studies shed light on male reproductive health. As such, findings showed lower levels of vitamin D in infertile women compared to healthy participants (55–64). For instance, over 83% of women with PCOS were found to be vitamin D deficient; however, 74% gained back normal values after 6 months of supplementation with metformin, vitamin D, and calcium. This therapy also adjusted the irregularity in the menstrual cycles and enhanced the follicular size and oocyte maturity of these women (57). Other authors even encouraged the optimization of serum calcium and vitamin D in sub-fertile women (58). Although adequate levels of vitamin D were associated with better endometrial thickness, not all proof suggested an increase in the rate of either chemical or clinical pregnancies (57, 59, 60). Only 2 studies agreed that prescribed vitamin D supplements might improve pregnancy rates (61, 65).

Moreover, other articles examined the changes in sexual hormones of supplemented infertile and fertile women. In one study, the researched group showed higher elevation of LH levels and lower elevation of FSH, estrogen, and progesterone, which was proposed to form a potential risk factor for infertility in general and miscarriage in particular (63). One RCT suggested a negative correlation between vitamin D supplementation and AMH serum levels in PCOS women while another showed that taking 50,000 IU of vitamin D per week for 12 wk elevated AMH serum levels (66). This improvement is attributed to vitamin D action on the gene promoter responsible for AMH expression without affecting the ovarian reserve (67). On the other hand, researchers witnessed a highly significant association between vitamin D and antral follicle count among 189 participants (68).

As for infertile men, all available publications measured the level of vitamin D after supplementation (with or without calcium or zinc) or without any supplementation and carried out reproductive hormone assessment and/or semen analysis for recruited study groups (69–84). It was shown that most vitamin D-deficient men were found to be infertile with impaired seminal fluid characteristics. As such, sufficient vitamin D levels reflected better reproductive functions.

As for FSH, researchers did not find a significant change in its levels but witnessed higher testosterone levels with higher serum vitamin D. Moreover, some mentioned a negative correlation between vitamin D and LH, whereas others suggested no impact of one other (61, 73). Several other studies also approved the lack of vitamin D contribution to all mentioned male sexual hormones along with both estradiol and progesterone (70, 77, 79, 82, 83). However, in an Iranian RCT, therapy with vitamin D3 did not affect the levels of total testosterone but increased the free testosterone present in the serums of the controls (83).

Lower sex hormone binding globulin (SHBG) was associated with low vitamin D concentrations, nevertheless, vitamin D3 intake significantly decreased the SHBG (79, 83). One study comparing the effects of vitamin D and L-carnitine supplements noticed an elevation in prolactin levels post-vitamin D administration (71). Several studies indicated a significant improvement in sperm motility as compared to 2 papers that disapproved of this suggestion and hypothesized that there was not enough evidence (69–71, 73, 74, 76, 77, 79–86).

Moreover, while some authors suggested that adequate levels of vitamin D played an efficient role in increasing the count and/or concentration in semen fluid and the percentage of morphologically normal spermatozoa, others showed that both characteristics, as well as the ejaculate volume, did not sufficiently change (69–71, 73, 74, 76–78, 80, 81, 83, 84, 87). Some also observed a bigger seminal volume among fertile men with higher serum levels of vitamin D or infertile men supplemented with vitamin D for 2 months (71, 76). A cohort study in 2022 addressed the effect of vitamin D on the embryological outcomes of infertile participants. The number of retrieved eggs, mature eggs, fertilization rate, and good transferred embryos did not show significant differences between sufficient, insufficient, and deficient groups. Only higher rates of implantation were attributed to vitamin D-sufficient participants (78). Concerning the DNA fragmentation index (DFI) of semen, controversial information was published: 2 studies found no correlation between vitamin D and DNA fragmentation, one found a significant difference between OAT men and normospermic, and one found a negative relationship between serum vitamin D and DFI (69, 78, 80, 81). As for the effect of calcium, low levels of Ca^2+^ were associated with decreased motility and increased FSH and LH, but no difference was observed in the levels of total testosterone, estradiol, or SHBG (79).

### Antioxidants

A significant portion of the literature on the risk factors that interfere with the reproductive system discussed antioxidants and oxidative effects. 3 studies assessed the dietary antioxidant intake of hundreds of infertile men. In Jordan, more than 40% seek alternative antioxidant supplementation, especially vitamin E, ginger, ginseng, and palmetto (88). Among the Iranian population, β-cryptoxanthin was positively associated with sperm motility and density while β-carotene and vitamin C were inversely related to sperm DNA fragmentation (89, 90).

Furthermore, β-carotene and selenium were shown to improve the ejaculate volume and sperm movement, explaining lower vitamin A, lycopene, and β-carotene levels and higher DFI in infertile men (91, 92). However, one study comparing OAT and fertile men did not find a significant difference in sperm motility, concentration, DFI, and total antioxidant capacity (TAC) (93). On the other hand, N-acetylcysteine (NAC) improved sperm quality post varicocelectomy (94) and supplements like alpha-lipoic acid, zinc sulfate, and ubiquinol enhanced sperm parameters, mainly the count, morphology, and motility (95–97).

Ubiquinol supplementation was suggested to interfere with the sexual hormone balance by decreasing the level of FSH and increasing the level of inhibin B in serum (96). CoQ10 was studied in various contexts: a 6 month-administration of 200 mg of CoQ10, alone or with 250 mg of glutathione, improved sperm motility and concentration, and CoQ10 with arginine boosted seminal volume, morphology, and sperm activity after 3 months (98, 99). A daily CoQ10 dose alone in OAT men also improved motility and concentrations, better catalase and superoxide dismutase activities, and a more enhanced TAC compared to baseline (100). In addition, lower CoQ10 levels in infertile men showed higher* miR378 *gene expression usually involved in the regulation of spermatogenesis (101). 2 Iranian publications evaluated the impact of multivitamins on semen. Androferti supplements (L-carnitine, vitamin C, CoQ10, B9, B12, zinc, selenium) increased the number of correctly shaped sperms and their concentration, but had no impact on their motility (102). However, a mixed therapy of vitamin E, vitamin C, and CoQ10 enhanced the motility, DNA integrity, and natural pregnancy rate (103). The antioxidant power of vitamin E and clomiphene citrate also improved semen parameters in infertile men (48). Several studies compared normal and asthenospermic or OAT men for their TAC and their malondialdehyde (MDA) levels. Although TAC was correlated with sperm parameters, MDA levels were not, yet both were linked to infertility (50, 104–107). Supplementation with vitamin D, E, Carob, probiotics, or honey intake along with physical activity led to increased TAC and decreased MDA and reactive oxygen species (47, 79, 78, 81, 108). Alpha-lipoic acid supplementation for 12 wk; however, increased both variables (76).

The effect of antioxidants on women's fertility was discussed in 5 studies. Melatonin was shown to improve embryo qualities and slightly increase chemical and clinical pregnancies without altering the expression of the mitochondrial adenosine triphosphate production (*MT-ATP6*) gene in the cumulus of the ovarian follicles between study groups (109).

Similarly, NAC or CoQ10 supplementation reduced the percentage of metaphase I and dysmorphic oocytes and increased the number of high-graded embryos (110, 111). On the other hand, one paper did not find a significant difference in the embryological outcomes of women given multivitamins and minerals (112). In another comparative study, no difference in leptin, prolactin, FSH, or LH levels was detected, except in women with PCOS taking 1800 mg of NAC alone or with metformin daily for 6 wk, leptin, LH and insulin levels decreased as compared to the placebo (112). In another study, infertility was correlated with higher Cu, 4-hydroxynonenal, and lipid hydroperoxide and lower Mg, Zn, and Se. Groups with either primary or secondary infertility had also lower superoxide dismutase, catalase, TAC, and glutathione (113).

### Other supplements 

Other supplements were studied for their effects on male and female fertility. 4 RCTs found that the daily administration of L-carnitine, alone or combined with tamoxifen, pentoxifylline, or vitamin D, improved the sperm count, concentration, motility, and morphology in infertile men (114–117). It also positively affected the outcomes of intracytoplasmic sperm injection, especially pregnancy rates (116–118). L-carnitine supplements increased testosterone and inhibin levels and decreased LH and FSH ones, altering the endocrine status of men (114). In women with PCOS, L-carnitine improved hormonal profiles and menstrual regularity compared to controls in a group of 72 participants (119). Alhussien et al. also suggested lower reactive oxygen species and apoptosis that increased the implantation rate (120).

A randomized trial divided 40 men into 2 groups: one received 2 g of spirulina, 220 mg of zinc sulfate, 500 mg of L-carnitine, and 50 mg of clomiphene daily for 12 wk, while the other group received a placebo. The semen fluid characteristics were indifferent; however, the intervention group had a 5% pregnancy rate while the placebo group had none (121). Another randomized controlled study on women with PCOS, given selenium or placebo for 8 wk, showed no effect of supplementation on the pregnancy rates (122).

This review outlined the significant impacts that dietary habits can have on the fertility of both men and women, as well as their impact on the success rates of ART.

For men, it was shown that a reduction in red meat, fatty foods, and whole grains and an increase in fish, poultry, low-fat dairy products, vegetables, and good fats, such as nuts positively affect sperm quality and quantity, thus improving fertility outcomes. However, for women, it was found that maintaining a healthy weight is as crucial as eating a healthy diet rich in fruits, vegetables, red and white meat, and nuts. Whilst the same recommendations apply to ART and IVF, studies are still inconclusive regarding all the food groups, but sticking to a traditional healthier diet prevents the risk of spontaneous abortion. Meat consumption was considered healthy for women but unhealthy for men, but the evidence is still inconclusive to generate any general recommendations. This conclusion on meat and the consumption of fruits, vegetables, and oils showed similar variations in international studies, as evidenced by a systematic review by Winter et al. which included 11 studies from multiple contexts including the USA, Japan, the Netherlands, Spain, Italy, China, and Greece. However, it was discovered that a high adherence to the Mediterranean diet (focused on plant-based food and healthy fats) may enhance the rates of live births and pregnancies by about one-third; upon excluding studies with a significant risk of bias, these rates rose to more than two-thirds (123), which also correlates with the findings of our review.

Furthermore, lipids hold a key role in the reproductive system. Based on the findings, it appears that taking omega-3 supplements could raise a woman's chances of getting pregnant as well as improving the quality of the sperm. These findings are aligned with international systematic reviews suggesting the positive correlation between omega 3-specifically, and fertility (124, 125). More precisely, a diet richer in omega-3 fatty acids is linked to better health and a lower chance of developing chronic illnesses (124), also suggesting that supplementation in omega-3 fatty acids improves fertility rates (125).

Overall, various vitamins and supplements were proven to have a significant impact on improving fertility by enhancing sperm and egg quality, hormonal balance, and antioxidant capacity, despite that the results were not consistent across all studies. Vitamins B9 and B12 were found to improve sperm parameters in infertile men and improve ART outcomes in women. An increased intake of preconception supplementary folate may improve a woman's chances of becoming pregnant and possibly carrying a pregnancy to term, according to the systemic review conducted in 2018 on folate and fertility endpoints, and the positive benefits of folate on fecundity and fertility were seen in multiple studies (2).

2 studies conducted in the USA and Spain found a positive correlation between the intake of vitamin C, E, β-carotene and the total sperm count, concentration, semen volume, and total progressive motility (126, 127). These results complement the positive correlation of vitamin C and E on men's infertility depicted in this review. On the other hand, while it was shown that supplementations improve menstrual cycles in women and decrease free testosterone in men, not all studies showed improved pregnancy rates and reproductive hormones. Because of the wide variety of outcomes, it is now difficult to draw firm conclusions from data about vitamin D and fertility.

This review identifies several key limitations. Across the EMR, dietary habits were primarily examined in Iranian infertile groups. Furthermore, the predominant use of observational designs rather than RCTs hinders the ability to establish causal relationships. Despite some disagreements on the effects of specific nutrient groups, similar major dietary patterns were identified. Additionally, 3 studies investigated the impact of daily food consumption on the embryological outcomes of IVF, focusing exclusively on Iranian couples. This highlights the need for a cross-cultural understanding of nutritional risk factors in infertility. Lipids were studied only once among men, and no interventional studies on lipid digestion for men or women seeking IVF treatment were found. Similarly, the review did not identify any RCTs assessing the administration of vitamin B to infertile men or vitamin C to infertile women. While vitamin D was extensively evaluated, results varied regarding its correlation with pregnancy rates and semen parameters. More research is needed to understand the impact of vitamin D on sexual hormones in men and AMH levels in women. Additionally, there was a lack of interventional studies focusing on the embryological outcomes of IVF patients taking vitamin D supplements. Limited information was available on calcium supplementation for infertile men and women. Antioxidants were mainly studied in both healthy and infertile men, but the variation in the DFI was not always significant, indicating the need for further studies. One study mentioned the potential impact of probiotics on men's fertility, more research, including studies recruiting both women and men, is necessary to verify the efficacy of probiotics.

The existing information on the effects of diet-related factors on human reproduction and assisted reproductive techniques remains controversial and inconclusive. Given the emerging negative associations with certain food patterns, larger and more thoroughly planned observational and clinical studies are essential. Proven evidence provides helpful daily recommendations that can enhance general knowledge and clinical practice.

Despite these limitations, the review underscores the importance of understanding dietary impacts on fertility and provides a foundation for future research. To enhance the reliability and applicability of findings, emphasis should be placed on lipids, vitamins (B, C, and D), calcium, and probiotics. Standardizing research designs and outcome measures will address inconsistencies and facilitate comparison across studies. Additionally, investigating the direct impact of dietary factors on embryological outcomes of IVF treatments, including semen quality, sexual hormones in men, and AMH levels in women, is crucial.

## 3. Conclusion

This review provides a comprehensive analysis of the impact of dietary habits and food supplementation on reproductive health in the EMR. The findings highlight distinct dietary patterns that either mitigate or exacerbate infertility risks, considering macro and micronutrients. The current evidence on ART risk factors within this region is insufficient. Studies unevenly describe potential risk factors, underscoring the need for further exploration across diverse populations.

##  Data Availability

Not applicable.

##  Conflict of Interest

The authors declare that there is no conflict of interest.
